# The impact of violence on Venezuelan life expectancy and lifespan inequality

**DOI:** 10.1093/ije/dyz072

**Published:** 2019-04-21

**Authors:** Jenny García, José Manuel Aburto

**Affiliations:** 1 French Institute for Demographic Studies (INED), Université Paris 1 Panthéon Sorbonne, Paris, France; 2 Interdisciplinary Centre on Population Dynamics, University of Southern Denmark, Odense, Denmark; 3 Max Planck Institute for Demographic Research, Rostock, Germany

**Keywords:** Causes of death, decomposition analysis, homicides, young male mortality, firearm-related deaths, cardiovascular revolution

## Abstract

**Background:**

Venezuela is one of the most violent countries in the world. According to the United Nations, homicide rates in the country increased from 32.9 to 61.9 per 100 000 people between 2000 and 2014. This upsurge coincided with a slowdown in life expectancy improvements. We estimate mortality trends and quantify the impact of violence-related deaths and other causes of death on life expectancy and lifespan inequality in Venezuela.

**Methods:**

Life tables were computed with corrected age-specific mortality rates from 1996 to 2013. From these, changes in life expectancy and lifespan inequality were decomposed by age and cause of death using a continuous-change model. Lifespan inequality, or variation in age at death, is measured by the standard deviation of the age-at-death distribution.

**Results:**

From 1996 to 2013 in Venezuela, female life expectancy rose 3.57 [95% confidence interval (CI): 3.08–4.09] years [from 75.79 (75.98–76.10) to 79.36 (78.97–79.68)], and lifespan inequality fell 1.03 (–2.96 to 1.26) years [from 18.44 (18.01–19.00) to 17.41 (17.30–18.27)]. Male life expectancy increased 1.64 (1.09–2.25) years [from 69.36 (68.89–59.70) to 71.00 (70.53–71.39)], but lifespan inequality increased 0.95 (–0.80 to 2.89) years [from 20.70 (20.24–21.08) to 21.65 (21.34–22.12)]. If violence-related death rates had not risen over this period, male life expectancy would have increased an additional 1.55 years, and lifespan inequality would have declined slightly (–0.31 years).

**Conclusions:**

As increases in violence-related deaths among young men (ages 15–39) have slowed gains in male life expectancy and increased lifespan inequality, Venezuelan males face more uncertainty about their age at death. There is an urgent need for more accurate mortality estimates in Venezuela.

**Key Messages**

- The recent upsurge in violence has slowed male life expectancy gains related to reductions in mortality from other causes (e.g. circulatory, infectious and respiratory diseases).
- Increasing lifespan inequality due to violence-related mortality corresponds to greater uncertainty among Venezuelan males about their age at death.
- Counteracting the eroding effect of violence, decreases in cardiovascular mortality and under-five mortality are the main drivers of increasing life expectancy in both sexes.
- Venezuela could improve its overall longevity and lower its lifespan inequality by reducing violent deaths.

## Introduction

Most Latin American countries experienced sizable improvements in health, living standards and longevity in the second half of the 20th century.^1^ In Venezuela, mortality declined progressively from 1930 onward,^2^ with life expectancy increasing from 54.9 years in 1950 to 74.2 years in 2013.^3^^,^^4^ These advances were driven first by a reduction in infant mortality;^5^ then by the postponement of death in adults; and, finally, by improvements in old-age mortality.^6^ However, gains in life expectancy have slowed for both sexes since the mid-1990s. While life expectancy increased by 3.8 years per decade between 1950 and 1990, gains since 1990 have fallen to 1.8 years every 10 years.^3^

This slowdown coincides with a continuous rise in levels of violence in Venezuela. At the beginning of the 1990s, an ‘epidemic’ of violence had already been identified.^7^^,^^8^ Homicides increased steadily and more than doubled between 1995 and 2009 (from 20.3 to 49.0 homicides per 100 000 inhabitants).^9^ By 2010, around 13% of all deaths were due to violence and injuries,^10^ and the country had the fourth-highest crude mortality rate from external causes in the world.^11^

Life expectancy is the most widely used indicator of population health, and it reflects the overall level of longevity of a population. In this study, we assess the contribution of violence to the recent slowdown in life expectancy gains in Venezuela. We also quantify the effect of violent deaths in an equally important dimension of health: the dispersion of ages at death, or lifespan inequality.^12^ Lifespan inequality is an indicator of how similar ages at death are, and has become an important public health topic alongside health inequalities.^13^ It is interpreted as a marker of heterogeneity in ages at death at the macro level^14^ and of survival uncertainty at the individual level.^15^^,^^16^ Since lifespan inequality is highly sensitive to premature mortality^15^ and homicides are concentrated at working ages, the net effect of the upsurge of violence is unknown. Studying life expectancy alongside lifespan inequality in this context may give policymakers a better understanding of the effects of violence on population health. The combination of the two indicators suggests that individuals’ decisions are based not only on their expected lifespan, but on their uncertainty about when they will die.^12^

Over the last decade, public institutions in Venezuela have been forced to follow a strict policy of secrecy. Since 2013, data sources have not been updated nor made publicly available.^17^ The government's constant denials that mortality is increasing, and particularly mortality due to external causes, has made it difficult to assess recent trends. The withholding of official homicide counts started well before 2013. The last official annual homicide data were made publicly available a decade earlier.^18^ Official data on homicides are traditionally generated by law enforcement authorities’ determination of the intent in each case. However, in the absence of law enforcement data, it is possible to use the statistical information produced by health authorities certifying the cause of death of individuals.^9^ To compensate for this lack of data, we corrected the death counts published by the Ministry of Health for the period 1996–2013.

Since the beginning of the 1990s, homicide mortality in Venezuela has been concentrated between ages 15 and 50, and has mainly affected males (10 times more males than females).^19^ We therefore hypothesize that deaths from homicides have contributed to the slowing of improvements in male life expectancy. As life expectancy and lifespan inequality are negatively correlated,^15^ we expect to observe a similar adverse effect on lifespan inequality reductions. We focused on the period after 1996 to test our hypotheses. During this period, life expectancy trends in Venezuela were affected not only by the upsurge in violence, but by large declines in deaths from circulatory diseases^20^ and the emerging importance of cancer and diabetes.^4^

## Data

We examined death counts from official mortality yearbooks reported by the Venezuelan Ministry of Health from 1996 to 2013.^21^ We used annual population estimates from the Venezuelan National Institute of Statistics^22^ as the denominator to compute age-specific death rates. For data availability and assessment, see Supplementary Material: 1. Data sources in Venezuela, pp. 2–5, available as Supplementary data at *IJE* online.

Causes of death were classified according to the 10th revision of the International Classification of Diseases (ICD-10) and grouped into: (i) circulatory diseases (e.g. heart diseases, hypertension, ischaemic heart diseases and cerebrovascular diseases); (ii) neoplasms; (iii) diabetes; (iv) homicides and other violent deaths (e.g. homicides, undetermined intent and legal intervention); (v) other external causes (e.g. traffic accidents, injuries and suicide); (vi) respiratory diseases; (vii) infectious diseases; (viii) digestive diseases; (ix) conditions originating in the perinatal period; and (x) remaining causes. For a detailed description, see Supplementary Material: 4. Cause of death classification Table 3, p. 15, available as Supplementary data at *IJE* online. To improve the accuracy of our estimates of violent deaths, we defined them as cases in which the death certificate stated that the cause of death was ‘homicide’ or a violent death of undetermined intent; see details in Supplementary Material: 1.2 Mortality data, p. 2, available as Supplementary data at *IJE* online.

To ensure data quality, mortality estimations were adjusted for under-reporting, age misreporting and ill-defined causes of death.^23^^,^^24^ Adjustment for the impact of under-reporting on mortality rates was undertaken by applying indirect estimation methods on data from the 1990, 2001 and 2011 population censuses. Specifically, the synthetic extinct generation and the General Growth Balance methods were combined^25^ to estimate adult mortality (Supplementary Material section 2.1.1 Adult mortality Coverage, pp. 7–9, available as Supplementary data at *IJE* online), and the Trussell variant of the Brass method^26^ was used to estimate infant mortality (Supplementary Material section 2.1.2 Infant mortality coverage, p. 9, available as Supplementary data at *IJE* online). A set of under-registration ratios was obtained by contrasting intercensal indirect estimation and directly estimated mortality rates. These under-registration ratios were linearly interpolated and extrapolated into the time frame of our analysis. We assumed that the rate of under-registration did not fluctuate greatly, but declined smoothly at a constant pace.

All data assessments and quality adjustments are detailed in the Supplementary Material: 2. Quality of content, pp. 9–12, available as Supplementary data at *IJE* online. Additionally, a robustness check of our life expectancy estimates and violence-related death rate was carried out using international sources in Supplementary Material: 3. Comparing with life expectancy estimations, p. 14, available as Supplementary data at *IJE* online.

## Analytical methods

Annual period life tables were constructed using standard demographic methods.^27^ From these, life expectancy and lifespan inequality were calculated with 95% empirical confidence intervals from bootstrapping with exponential distribution with piecewise constant rate.^28^ The latter was measured by the standard deviation of the age-at-death distribution (σ).^29^ Changes in both indicators during the study period were decomposed based on a continuous change model.^30^ An advantage of this method is that it assumes that covariates change gradually (Supplementary Material 7. Description of the decomposition method, p.19, available as Supplementary data at *IJE* online). Through decomposition, we dissected contributions (in years) to changes in life expectancy and lifespan inequality by each cause of death at each age.^31^ For further details, see Supplementary Material: 6. Brief description of the lifespan variation indicator, pp. 15–16, available as Supplementary data at *IJE* online.

Although several lifespan inequality indicators exist (e.g. Gini coefficient, life years lost, variance), the high degree of correlation between them suggests that our main results would be consistent with those obtained by another indicator.^29^ In addition, by using the standard deviation, we ensure the comparability of lifespan inequality with life expectancy because both are expressed in years. These indicators were chosen because they are easy to understand, interpret and decompose, and thus allow us to quantify changes in age- and cause-specific mortality over time.

## Results

**Figure 1:**
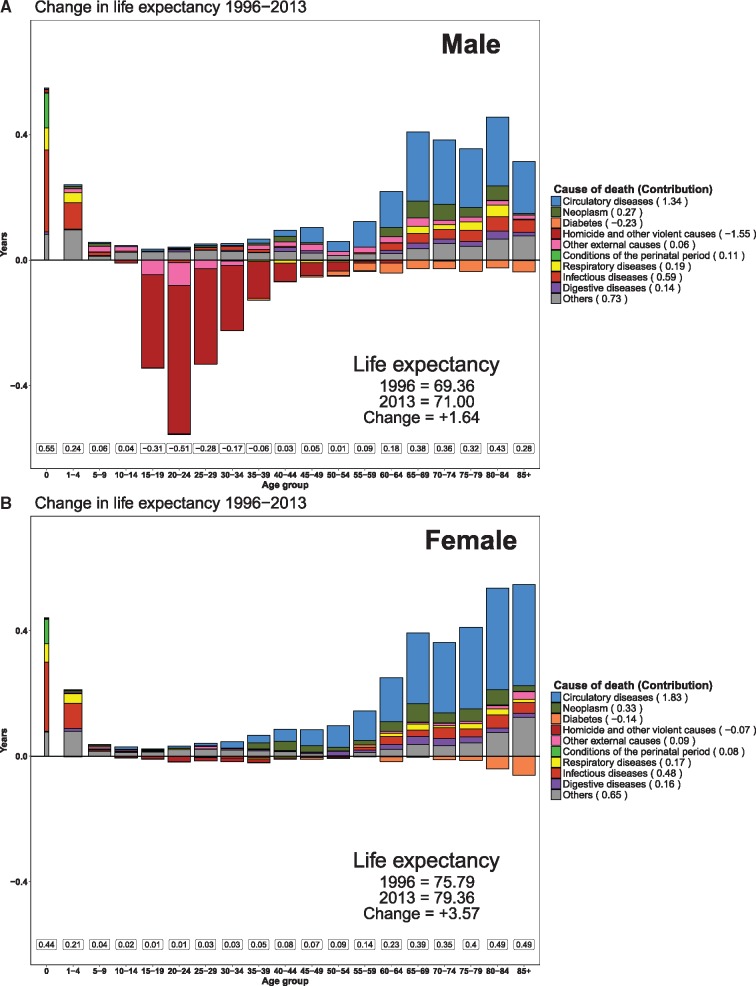
Top (Male) and bottom (Female) shows the age- and cause-specific contributions to changes in life expectancy between 1996 and 2013. Positive (i.e. life expectancy increases) and negative (i.e. life expectancy losses) contributions by cause of death are indicated by the bars. Additionally, the overall contributions by age group are shown in the boxes at the bottom, and the total contributions by cause of death are indicated in parentheses in the legend.

Between 1996 and 2013, male life expectancy increased 1.64 [95% confidence intervals (CI): 1.09–2.25] years [from 69.36 (68.89–69.70) to 71 (70.53–71.39) years)], while female life expectancy increased twice as much [from 75.79 (75.38–76.10) to 79.36 (78.97–79.68) years], gaining 3.57 (3.08–4.09) years. Differences in mortality reductions led to an increase in the sex differential in life expectancy from 7.19 years in 1996 to 8.76 years in 2013, see Figure 1.

The largest gains in life expectancy for both females and males occurred at ages below 1 year and above 55 years. Infant mortality accounted for 13% (+0.44 years) and 33% (+0.55 years) of gains in life expectancy for females and males, respectively. The gains were mostly due to reductions in deaths from conditions originating during the perinatal period, and from respiratory and infectious diseases. At older ages, decreasing mortality from circulatory diseases led to life expectancy gains of 1.83 years for females and 1.34 years for males. However, these gains were offset by increasing mortality from diabetes and violence. Diabetes mortality, which mostly affected the population over age 50, was associated with decreases in male and female life expectancy of 0.23 and 0.14 years, respectively. Meanwhile, homicide was the cause of death that had the largest negative impact on male life expectancy (–1.55 years), whereas the impact of this cause on female life expectancy was negligible (–0.07 years). Life expectancy losses due to homicide were concentrated in men between the ages of 15 and 50.

**Figure 2:**
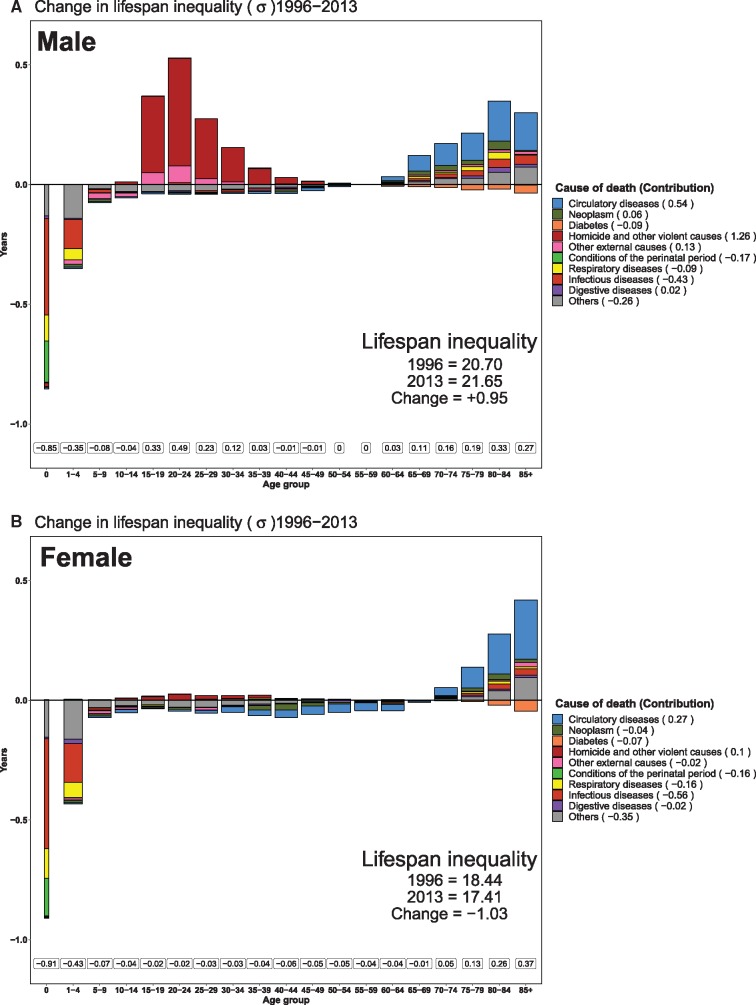
Top (Male) and bottom (Female) shows the age- and cause-specific contributions to changes in lifespan inequality. Major improvements in under-five mortality (mainly due to fewer deaths from perinatal conditions) and in mortality from infectious and respiratory diseases together led to decreases in lifespan inequality of 1.20 years in males and of 1.34 years in females. However, these improvements were offset by the surge in male homicide mortality at young ages (15–45). Homicides and other violent causes led to an increase in male lifespan inequality of 1.26 years. Lifespan inequality also increased due to improvements in cardiovascular mortality at ages 60 and older (blue bars).

Between 1996 and 2013, lifespan inequality decreased 1.03 (–2.96 to 1.26) years [from 18.44 (18.01–19.00) to 17.41 (17.30–18.27) years] for females, but increased 0.95 (–0.81 to 2.89) years [from 20.7 (20.24–21.09) to 21.65 (21.34–22.12)] for males, see Figure 2.

## Discussion

Between 1996 and 2013, the impact of violent deaths on male life expectancy in Venezuela was substantial (–1.95 years). The increase in homicides offset improvements in mortality from other causes such as cardiovascular diseases, and led to an unprecedented increase in lifespan inequality (0.95 years). Recent homicide trends suggest that conditions will not improve, and may even deteriorate.

When studying Latin American mortality, the first challenge is undoubtedly insufficient data quality and coverage. During the 20th century, Venezuela’s vital statistics system was more robust than those of most Latin American countries.^32^ Coverage improvements in mortality registry data continued during the first decade of the 21st century.^33^^,^^34^ However, the government’s recent policy of strict secrecy has made it difficult to access data, and thus to assess mortality trends. Here, we contribute to the existing literature on Latin American mortality by overcoming those limitations and producing robust estimations of life expectancy, lifespan inequality and violence-related deaths by using established powerful methodologies to adjust for under-registration during the period of our analysis.

Additionally, in the interests of accuracy, we identified violent deaths by grouping death counts in which the underlying causes of death could include not just ‘assault’, but also an ‘event of undetermined intent’ or ‘legal intervention and operations of war’. We used this approach because homicides are not usually counted as such in the mortality yearbooks if the intent was not legally determined before the death certificate was produced. The legal determination of intent depends on the findings of the police investigation, and not on the actual occurrence of the violent act. Our strategy therefore enabled us to estimate death counts that correspond more closely with official data on annual homicides (released up to the year 2003) and unofficial figures reported by national non-governmental organizations (Supplementary Material: 1.1 Mortality data, pp. 2–3, available as Supplementary data at *IJE* online).

Our findings show that improvements in both life expectancy and lifespan inequality were mostly driven by reductions in under-five mortality. Decreases in deaths from cardiovascular diseases also contributed greatly to increasing life expectancy. These results are in line with the trends we would expect to observe during the second cycle of the health transition.^35^ The main development that ran counter to such positive changes was the upsurge of violence, which had such a large detrimental impact on Venezuelan males that all of their gains in life expectancy from reductions in deaths from other causes were eroded. As a result of this trend, improvements in life expectancy have slowed, and as we show, lifespan inequality has even increased. Males in Venezuela are not just dying earlier on average; they are facing greater uncertainty about their eventual time of death due to the threat of premature mortality by violence. Globally, there are very few national-level examples of periods in which life expectancy stagnated or decreased while lifespan inequality increased. During a period of life expectancy stagnation that occurred in the 1960s and 1970s in the former Soviet Union, over half of the time the yearly changes in life expectancy and lifespan inequality occurred in the same direction due to mortality deterioration at young ages.^36^ Thus, ours is the first study to document an effect of high homicide rates on increasing lifespan inequality in tandem with stagnation in life expectancy at the national level. In the United States, life expectancy has stagnated, but lifespan variation has increased among males as the opioid epidemic has spread.^13^

This excess young male mortality is a recent phenomenon. Throughout the 1980s, the homicide rate in Venezuela was relatively low; i.e. it was close to the levels observed in countries like Costa Rica, at around eight per 100 000 inhabitants.^37^ However, the social and economic upheavals of that decade led to changes in the prevalence of violence in Venezuela.^38^ Prior to that period, violence in Venezuela was largely related to political conflict, and was rarely the subject of explicit attention. Thereafter, violence became associated with the urban agglomerations and ‘slums’ that sprang up as the country experienced rapid urbanization and high levels of rural–urban migration. This shift in prevalence reflects the increase in social inequality and the fragile legitimacy of the state, together with a genuine ‘culture of violence’^37^ that has intensified in most Latin American countries.

Three major violent events preceding our time frame have been identified as tipping points: the popular uprising against price increases known as ‘El Caracazo’ (1989) and two attempted coups (1992 and 1993).^19^^,^^39^ These events led to a doubling of the homicide rate, and since then violence has not been contained. To put these trends in perspective, the homicide rates in Venezuela in 2012 (53.7 per 100 000 inhabitants) were higher than those of Latin American countries that have experienced undeclared civil wars in recent decades,^7^ such as El Salvador (41.2), Guatemala (39.9) and Colombia (30.8). In 2010, the violent death rate was higher in Caracas (80.6) than it was in war-torn Iraq (54.6).^11^ Legally declared homicides, ‘extra-judicial executions’ by criminal organizations, police brutality and violent deaths of undetermined intent were the most common causes of these violent deaths.^40^ Similar results have been reported for Mexico, where life expectancy stagnated and lifespan inequality increased in some regions, due to increases in homicide mortality related to the war on drugs.^41–43^

The uniqueness of the Venezuelan experience could be linked to the combination of two circumstances: rapidly rising wealth and surging homicide rates. Unprecedented increases in national income per capita, which enabled the wider distribution of wealth at the individual level, occurred at the same time as homicide rates were rising sharply.^44^ Most studies have viewed income inequality as a social determinant of ill health and violence,^45^^,^^46^ and the evidence suggests that in most countries inequality is associated with higher rates of homicides and other crimes.^47^^,^^48^ The Venezuelan case challenges assumptions about this relationship. Since the early 2000s in Venezuela, indicators of poverty and inequality have decreased Supplementary Material: 8. Wealth and inequality in Venezuela, p. 19, available as Supplementary data at *IJE* online, whereas rates of kidnapping and of other crimes have increased Supplementary Material: 9. Crime in Venezuela, p. 20, available as Supplementary data at *IJE* online. This paradox may be attributable to the combined effects of extraordinarily large oil revenues and the failure of the state to guarantee the security of its citizens.^49^

Our results underscore the impact of violence on longevity and on lifespan inequality in Venezuela. The country could increase its overall longevity and decrease its lifespan inequality simply by focusing on a clear public health target of reducing homicides. To reverse the detrimental effects of violence, new public health interventions are needed. To reduce homicides, policies that disarm the civil population and effectively control the legal use of arms should be implemented. Most of the homicides reported in this analysis were committed with firearms, which are widely available in the country. The share of firearm-related deaths in Venezuela more than tripled between 1996 and 2013, from 1.8% to 6%. Furthermore, between 2003 and 2007, Venezuela was second among South American countries and 17th in the world in terms of military spending increases.^50^^,^^51^ In a context of institutional weakness, the supply of weapons in Venezuela has flooded a black market in which local police and armies have become the main weapons smugglers.^52^

At an official level, the Venezuelan government has implemented various policies aimed at stopping violence and criminal activities, including legal and structural reforms of all existing local police forces (2006), the establishment of a unique national police force (2009), and the creation of a presidential commission to control firearms and weapons (2011).^53^ As our results show, these efforts have been insufficient, not only because of the structural weaknesses of Venezuelan public institutions^44^ and their high levels of corruption, but because of the inconsistent (if not contradictory) attitude of the government regarding the use of violence.^54^ When we compare the situation of Venezuela with that of other countries in the region during our period of analysis, we see that Colombia and Venezuela not only experienced opposite trends, but almost swapped their homicide rates. From 2000 to 2012, Colombia managed to decrease its incidence of violence from a higher starting point (66.5 to 30.8 per 100 000 inhabitants) than that of Venezuela (32.9 to 53.7 per 100 000 inhabitants).^9^ These inverse outcomes are products of different institutional approaches to social control.^44^ While Venezuela’s institutions were experiencing a process of systematic annihilation, Colombia strengthened the credibility of its institutional mechanisms of access to justice.^55^

The future of Venezuela does not seem promising. Outbreaks of political violence have intensified in recent years, and the steady militarization of the police could lead to further increases in the prevalence of violence. Unfortunately, the additional effects of acute forms of political and socio-economic disintegration on mortality trends cannot be updated at this point in time. However, our findings suggest that male life expectancy trends are likely to shift from stagnation to decline, and that as a result, life expectancy and lifespan inequality will be negatively correlated.

## Funding

There was no funding for this study. The authors had full access to all data in the study and had final responsibility for the decision to submit for publication. The English editing of this manuscript was funded by European Research Council grant 716323.

## Acknowledgements

We thank Jacques Vallin (Emeritus Researcher at the Institut National d'Études Démographiques, Paris, France), and the two anonymous reviewers for helpful comments on the manuscript. The authors also thank to their home institutions for their support.

**Conflict of interest:** None declared.

## Supplementary Material

dyz072_Supplementary_Data
