# Supplementary material for: The impact of violence on Venezuelan life expectancy and lifespan inequality
Source: Int J Epidemiol. 2019 Apr 21;48(5):1593–601. doi: 10.1093/ije/dyz072 (PMC6857745; doi:10.1093/ije/dyz072)
Supplement: dyz072_Supplementary_Data [file dyz072_supplementary_data.docx]

**‘The impact of violence in Venezuelan life expectancy and lifespan inequality’**

**Supplementary material**

Authors: Jenny García ^a^ & José Manuel Aburto ^b^

Author affiliations:

^a^ Institut national d’études démographiques (INED), Université Paris 1 Panthéon Sorbonne, F-75020 Paris, France

^b^ Interdisciplinary Centre on Population Dynamics, University of Southern Denmark, Odense 5000, Denmark

Content

[1. Data sources in Venezuela 2](#_Toc531701509)

[1.1 Mortality data 2](#_Toc531701510)

[1.2 Population estimation 4](#_Toc531701511)

[1.3 Births 5](#_Toc531701512)

[2. Mortality data quality 7](#_Toc531701513)

[2.1 Coverage assessment 7](#_Toc531701514)

[2.1.1 Adult mortality Coverage 7](#_Toc531701515)

[2.1.2 Infant mortality coverage 9](#_Toc531701516)

[2.2 Quality of content 10](#_Toc531701517)

[2.2.1 Missing information 10](#_Toc531701518)

[2.2.2 Age and sex declaration 12](#_Toc531701519)

[2.2.3. Ill-defined causes of deaths 13](#_Toc531701520)

[3. Comparing with life expectancy estimations 14](#_Toc531701521)

[4. Causes of death classification 15](#_Toc531701522)

[5. Trends in violent causes of death 15](#_Toc531701523)

[6. Brief description of the lifespan variation indicator 15](#_Toc531701524)

[7. Description of the decomposition method 19](#_Toc531701525)

[8. Wealth and inequality in Venezuela 19](#_Toc531701526)

[9. Crime 20](#_Toc531701527)

[Reference 21](#_Toc531701528)

**List of graphs**

[Graph 1. Death counts by different official data source, 1990-2013. 2](#_Toc530758351)

[Graph 2. Death counts due to violence in Venezuela, different sources, 1990-2017. 3](#_Toc530758352)

[Graph 3. Venezuelan population estimations by sex (by thousands), age group and source 5](#_Toc530758353)

[Graph 4. Life expectancy estimations using corrected and uncorrected rates 8](#_Toc530758354)

[Graph 5. Venezuelan infant mortality rates by different method and data sources, 1996-2013 10](#_Toc530758355)

[Graph 6. Percentage of VHM’s death counts with missing information, 1996-2013. 11](#_Toc530758356)

[Graph 7. Age exagerattion ratios of death counts, 1996-2013. 11](#_Toc530758357)

[Graph 8: VHM’s death counts sex ratios, 1996-2013. 12](#_Toc530758358)

[Graph 9. Percentage of Ill-defined causes of death in VHM’s death counts, 1996-2013. 12](#_Toc530758359)

[Graph 10. Life expectancy at birth, comparison with different estimations 13](#_Toc530758360)

[Graph 11. Age-specific homocide rates by sex in Venezuela 15](file:///Z:\Jenny\Ponencias\Externas%20Venezuela\Externas%20Venezuela\Supplemental_material.docx#_Toc530758361)

[Graph 12. Age-specific violent male deaths by source, 1996-2013 16](file:///Z:\Jenny\Ponencias\Externas%20Venezuela\Externas%20Venezuela\Supplemental_material.docx#_Toc530758362)

[Graph 13. Age-specific violent female deaths by source, 1996-2013. 17](file:///Z:\Jenny\Ponencias\Externas%20Venezuela\Externas%20Venezuela\Supplemental_material.docx#_Toc530758363)

[Graph 14. Annual Gross Domestic Product (GDP) per capita and Gini coefficient, 1996-2013 18](#_Toc530758364)

[Graph 15. Percentage of population living in extreme poverty and population declaring not to have enough incomes,1996-2013 19](#_Toc530758365)

[Graph 16. Victimization rate and number of reported kidnapping cases 19](#_Toc530758366)

**List of Tables**

[Table 1. Venezuelan birth counts by different sources, 1996-2013 6](#_Toc530758377)

[Table 2:Death counts inter-census coverage estimation by death distribution methods 8](#_Toc530758378)

[Table 3. Causes-of-death groups and ICD codes 14](#_Toc530758379)

# **1. Data sources in Venezuela**

To calculate life expectancy it is necessary accurate mortality rates. To pursue this, we corrected death and birth counts and population older than one year old exposed to the risk of death at mid-year for the period 1996-2013. The first challenge was to select the most reliable data source. We describe next, the existing demographic data sources and estimations in Venezuela; we compared them and specified our selection and correction process. Later on, we put in perspective our estimations with those coming from international organizations such as World Health Organization (WHO), Latin American Mortality Database (LAMBdA) and Global Burden of Disease (GBD).

**1.1 Mortality data**

In Venezuela, there are only two official mortality and demographic data producers: Venezuelan Ministry of Health (VMH) and the Venezuelan National Institute of Statistics (VNIS). Historically, death counts reported by VMH have better coverage in comparison with VNIS (Graph 1). This is because official death counts published by the VMH as mortality yearbooks^(^[^1^](#_ENREF_1)^)^ -and reported by WHO - come from medical certificates. While VNIS’s death counts^(^[^2^](#_ENREF_2)^)^ come from registered death certificates, which involve an extra step for the deceased’s family members.

Deaths with medical certification increased significantly during the 70’s in Venezuela, up to 90% of total deaths had been certificated. This increase continued and, at the beginning of the 2000’s, only 0.3% of deaths lacked medical certification.^(^[^3^](#_ENREF_3)^)^ Even more, annual VMH’s reported deaths had good data quality: at the beginning of the 90’s, proportion of under-coverage was already estimated in less than 10% ^(^[^4^](#_ENREF_4)^)^ and continuous improvements placed it around 2.4% during the first decade of the 21^st^ century.^(^[^3^](#_ENREF_3)^,^ [^5^](#_ENREF_5)^)^

Graph 1. Death counts by different official data source, 1990-2013.

PDF file versions of mortality yearbooks until year 2013 were available online on VMH’s official website. Since then, a strict secrecy policy has ruled public institutions regarding all mortality and health data ^(^[^6^](#_ENREF_6)^,^ [^7^](#_ENREF_7)^)^  and data sources are no longer updated nor publicly available. The secrecy policy has been even stronger on official homicide data. Systematically, specialized data sources on the topic have been disappearing from public access since the beginning of the twenty-first century. The Venezuelan Bureau of Scientific and Criminal Investigations (VBSCI) used to release official data on annual homicides. This information has no longer been publicly available since 2003.^(^[^8^](#_ENREF_8)^)^

Some national non-government organizations published unofficial homicides figures with the aim to uncover the true impact of the violence on ordinary Venezuelans. The Venezuelan Observatory of Violence (VOV) gathers the most regular published data since 2008. Graph 2 summarizes VOV and VBSCI homicide counts, as well as our violent deaths counts grouping on VMH and Pan-American Health Organization (PAHO) data.

PAHO’s homicides estimations are based on a standardized statistical model used to produce data on all causes of death, which provide only figures on intentional homicides. ^(^[^9^](#_ENREF_9)^)^ The same happens with VMH data, reason why we grouped as violent deaths those in which the death certificate stablished “Assault” (X85-Y09) normally assumed as homicide, but also “Event of undetermined intent” (Y10-Y34) and “Legal intervention and operations of war” (Y35-Y36). This is important because some homicides are usually not counted if the intent is not legally determined.

Graph 2. Death counts due to violence in Venezuela, different sources, 1990-2017.

Source: VBSCI = LACSO Briceno Leon, 2006, VOV= Venezuelan Observatory of Violence annual reports. Uncorrected violent death (ICDX: X85-Y09, Y10-Y34, Y35-Y36) from Venezuelan Ministry of Health (VMH) and Pan-American Health Organization (PAHO): Health Information Platform for the Americas (PLISA) <http://www.paho.org/data/index.php/en/indicators-mortality/mnu-lcd-en.html?showall=&start=2>,

**1.2 Population estimation**

The updating of the Venezuelan population estimation and projections 1950-2050 by sex and age groups were carried out in 2016 by VNIS in collaboration with ECLAC’s population division. VNIS has reviewed and updated their long-term national population estimates and projections taking into account the latest census rounds (2001, 2011).^(^[^10^](#_ENREF_10)^)^ PRODEM (DEMographics PROjections) was used to elaborate official population estimations and projections in the context of each census round results. The method applied is the demographic component method that corresponds to an adaptation of the projections programs ABACUS and RUP (Rural-Urban Projections) developed by the United Nations Population Division. ^(^[^11^](#_ENREF_11)^)^

The biggest challenge experienced when estimating population is always related to migration estimations, especially in countries where migration may be important. Lack of continuous population registration hinders accurate estimations for return migration or migration flow. In Venezuela’s case, the inter-census period 2001-2011 did not show significant international migration flows, neither the period used as reference in this analysis (2013). According to United Nations Department of Economic and Social Affairs migrant stock estimations ^(^[^12^](#_ENREF_12)^)^ in 2000 international emigrants were 317,323 and 2010, they raised to 550,420. On the other side, international immigration stock in Venezuela was 1,013,663 in 2000 and 1,331,488 in 2010.

Household and population censuses in Venezuela have been regular since 1950, each ten or eleven years a census has been conducted by the VNIS. The unit of analysis has been restricted to the household and always has followed a *De jure* definition (enumeration of individuals as of where they usually reside). The three lasts census 1990, 2001 and 2010 are available online in the ECLAC’s REDATAM platform.^(^[^13^](#_ENREF_13)^)^ “REtrieval of DATa for small Areas by Microcomputer”- REDATAM is used by Latin American National Statistical Offices, either for data processing or online data consulting via REDATAM Webserver. ECLAC’s permanent assistance to Latin American countries has resulted in standardized procedures. Moreover, ECLAC makes available on line information on Venezuelan demographic patterns in several comparative thematic databases such as: the International Migration in Latin America database (IMILA),^(^[^14^](#_ENREF_14)^)^ the Internal Migration in Latin America and the Caribbean database (MIALC)^(^[^15^](#_ENREF_15)^)^, Indigenous and Afro-descendant Population database (PIAALC)^(^[^16^](#_ENREF_16)^)^ and Urbanization and Spatial Population Distribution database (DEPUAL)^(^[^17^](#_ENREF_17)^)^ all publicly available.

Direct evaluations on census 1990 and 2001 data were carried out by VNIS through post-enumeration surveys as well as indirect evaluation on census 2011. Results showed an omission of 9.1% (1990), 7.8% (2001) and 6.5 % (2011).^(^[^18^](#_ENREF_18)^)^ Omission has been concentrated in young male population and under five-year population. Both omission patterns have been considered to produce VNIS population estimations.

At the international level, other population estimations may be found for the same period. United Nations Economic Commission for Latin America and the Caribbean (ECLAC) produced and published population estimations ^(^[^19^](#_ENREF_19)^)^ in 2016. These estimations cover the period 1950 to 2100. ECLAC’s long–term figures were updated using household and population censuses data. The latest census gleaned in 2011^(^[^20^](#_ENREF_20)^)^ allowed ECLAC to review recent trends in fertility patterns. Other source producing population estimations is the World Health Organization. All estimations are presented in Graph 3. ECLAC’S estimation and VNIS show similar patterns and levels for all age groups. The main difference between these estimations and WHO’s are seen in the youngest age group (0 to 14 years old), and in the oldest age group (more than 55 years). WHO tents to over-estimate population in younger ages and females over 75 years, while under-estimating males over 55 years in comparison with ECLAC’s and VNIS’s. Consequently, the total number of population estimate each year is larger in WHO’s estimations. We decided to use VNIS annual estimation to produce mortality rates.

## **1.3 Births**

Official birth counts were traditionally published by the Venezuelan National Institute of Statistics (VNIS) and Venezuelan Ministry of Health (VMH). Official VMH birth counts based on birth certificates are only available until the year 2009. ^(^[^21^](#_ENREF_21)^)^ No official technical document was found related to how these estimates were produced or corrected. On its side, VNIS continued publishing birth counts on its official website until the year 2012,^(^[^22^](#_ENREF_22)^)^ however, the only time referenced on this data is the year of registration and not the year of occurrence of the event, reason why they cannot be included in this analysis.

Considering the lack of information, we relied on estimated birth counts produced by international organizations. the United Nations Economic Commission for Latin America and the Caribbean (ECLAC) published in 2016 birth counts for all Latin American countries ^(^[^23^](#_ENREF_23)^)^ during the period 1950 to 2100. These long–term figures are updated considering household and population censuses data and vital registered statistics. The latest census collected in 2011 allowed ECLAC to review recent trends in fertility patterns. We also compared birth counts estimations coming from different international organization, such as WHO and GBD, all sets are displayed in Table 1.

Graph 3. Venezuelan population estimations by sex (by thousands), age group and source


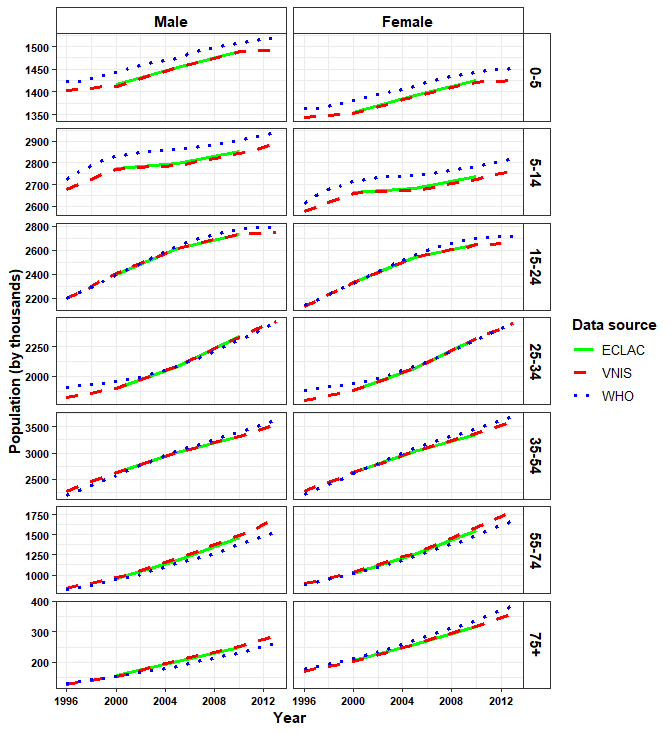


In comparison, birth counts estimated by WHO and GBD are larger than those estimated by ECLAC for the whole period. We chose to use birth counts produced by ECLAC because their estimations are based on the most recent corrected census data.

Table 1. Venezuelan birth counts by different sources, 1996-2013

| **Year** | **VMH** | **VNIS** | **ECLAC** | **WHO** | **GBD** |
| --- | --- | --- | --- | --- | --- |
| 1996 | 497975 |  | 569490 | 575937 |  |
| 1997 | 516636 |  | 569815 | 576664 |  |
| 1998 | 501808 |  | 570139 | 577771 |  |
| 1999 | 527888 |  | 570464 | 579275 |  |
| 2000 | 544416 | 544416 | 570789 | 581150 | 572980 |
| 2001 | 529552 | 529552 | 571114 | 583322 |  |
| 2002 | 492678 | 492678 | 571438 | 585638 |  |
| 2003 | 555614 | 555614 | 571763 | 587952 |  |
| 2004 | 530565 | 637799 | 572088 | 590172 |  |
| 2005 | 585655 | 665997 | 572413 | 592242 | 595790 |
| 2006 | 588500 | 646225 | 572738 | 594141 |  |
| 2007 | 591345 | 615371 | 573062 | 595878 |  |
| 2008 | 594191 | 581480 | 573387 | 597438 |  |
| 2009 | 594300 | 593845 | 573712 | 598768 |  |
| 2010 |  | 591303 | 574037 | 599805 | 603610 |
| 2011 |  | 615132 | 574361 | 600493 |  |
| 2012 |  | 619530 | 574686 | 600811 |  |
| 2013 |  | 597902 | 575011 | 600744 |  |

**2. Mortality data quality**

Determining the accuracy of the data is a prerequisite to any attempt to achieve reliability on estimations. Biased estimations are consequence of both inaccuracies in the data and errors in the assumptions involved to produce the estimates. Registered vital system data in Venezuela have historically had coverage and quality problems. Even when multiple of them have been overcome before and during the period of our analysis, a remaining proportion of under-registration in the data, misreporting age, and ill-defined causes of death persist. In order to determine and correct these errors, 1) we applied indirect mortality estimations to both infant and adult mortality as a complementary tool to evaluate our death counts; and 2) We assessed the data to determine the proportion of missing information, sex preference, age exaggeration and proportion of ill-defined causes of death.

**2.1 Coverage assessment**

We used indirect methods based on crosschecking against censuses to evaluate the coverage of VMH’s death counts estimations. We calculated coverage of adult and infant death counts separately using household and population censuses from 1990, 2001 and 2011.

### **2.1.1 Adult mortality Coverage**

There has been a consensus on the pertinence of using deaths distribution methods on Latin American mortality estimations,^(^[^24^](#_ENREF_24)^)^ mainly: the General Growth Balance method (GGB), the Synthetic Extinct Generations method (SEG) and a recent combination of both ^(^[^25^](#_ENREF_25)^)^ to determine the coverage by sex. Here we applied the combination of both methods. With these methods, a single estimation is obtained for each inter-census period: 1990-2001 and 2001-2011. Results came as proportions of death coverage that later on were linearly inter-extrapolated into a yearly coverage factor for the period 1998 to 2013, and applied to all deaths except for infants.

SEG method is based on the extinct generations method in which the number of people aged *x* at some past time can be estimated by accumulating all deaths in the population at ages *x* *and older,* when the last member of the cohort has died, leaving the entire cohort extinct. If registration of deaths is accurate, the estimated population $\hat{N}$ age *x* at time *t* is equal to the estimated population age *x+5* plus the number of actual deaths times the effect of growth rate $r_{x}$ Thus,

$\hat{N}_{x}= \hat{N}_{x+5}\exp\left( 5*5r_{x} \right)+5Dx\exp(2,5* 5 r_{x})$ (1)

For more precise growth estimation $5r_{x}$, we used the SEG variant including migration and growth rates for each age group (_5_NM_x_) as well as the differential omission coefficient ($\delta)$ on census data, then:

$5r_{x}=\frac{\ln\left( \frac{5Nx\left( t2 \right)}{5Nx(t1)} \right)}{t2-t1}-\frac{5NMx}{\left( t2-t1 \right)\left( 5Nx\left( t1 \right)*5Nx\left( t2 \right) \right)^{\frac{1}{2}}}+\delta$ (2)

The differential omission coefficient ($\delta)$ on census data was estimated using GGB method. This method is a variant of the Brass Growth Balance method. It comes from the idea that entries of each age group will be equal to the sum of outputs of each age group, being both linearly related. Since the relationship between the rates of input and output is linear, estimation of coverage (*c*) and differential omission coefficient ($\delta)$ are equal to the slope of the line derived from the points N_(x)_ / N_(x +)_ and D_(x +)_ / N_(x +)_. The differential omission coefficient ($\delta)$ is obtained comparing estimated omission on both census counts $\frac{k_{1}}{k_{2}}$

$\frac{N_{(x)}}{N_{(x+)}} -r_{\left( x+ \right)}= \frac{1}{t}*ln\left( \frac{k_{1}}{k_{2}} \right)+ \frac{\left( k_{1}+k_{2} \right)^{\frac{1}{2}}}{C}*\frac{D_{(x+)}}{N_{(x+)}}$ (3)

Each method has advantages and limitations, either way some requirements are constant: 1) the completeness of the reporting of deaths has to be the same for all ages and 2) there is no misreporting of age. The reason of these requirements is that none of the methods is designed to correct the population or death structures, they just adjust the level of estimated mortality. The combination of both methods allows producing mortality rates estimations, corrected from under-registration of death and isolated -as possible- of under-enumeration in population effects.

Migration data ($5NMx$ ) included in the indirect methods to estimate mortality rates came from Household and Population censuses (international immigration), and United Nations Department of Economic and Social Affairs (2015) census round worldwide (international emigration). To estimate the structures of the emigration stocks, we combined the total emigration stock by sex, with the proportional distribution of Venezuelan living in United States, for being this country the main destiny of attraction (37% of Venezuelan emigrants in 2010 lived in Unites States). Our assumption is that the structure found in the main destiny could represent population structure of all emigrants. Once stocks are estimated for each census round, it is possible to get the net inter-census migration flow.

$Net e^{F}=e_{(t+n)}^{F} - e_{\left( t \right)}^{F}+\left( \frac{n}{2}*\left( e+e_{(t+n)}^{F} \right){*m}^{F} \right)$ (4)

Where, e^F^ represents the total number of Venezuelan emigrants on the time *t* and *t+n,* and m^F^ is an estimate of the crude mortality rate of the population in the country of origin. We used mortality rates estimated by ECLAC’s for the period. Once inter-census international emigration flows are produced, net inter-census migration by age group and sex is estimated as:

$Net {{}_{5}M}_{x}$ $=\left( \left( Net {{}_{5}i}_{x}+\left( \frac{n}{2}*\left( e+e_{(t+n)}^{F} \right){*m}^{F} \right) \right)- Net {{}_{5}e}_{x} \right)$ (5)

Where,${{}_{5}i}_{x}$ represents the number of inter-census immigrants aged *x* to *x+5* at the time *t* and ${{}_{5}e}_{x}$emigrants.

We used the R package DDM developed by Tim Riffe, Everton Lima and Bernardo Queiroz in 2017 to apply indirect for adult mortality methods. Results are displayed in Table 2.

*Table 2. Death counts inter-census coverage estimation by death distribution methods*

| Sex | Inter-Census period | GGBSEG | Age group | | $\delta$ |
| --- | --- | --- | --- | --- | --- |
|  |  |  | Lower | Upper |  |
| Male | 1990-2001 | 0.9347 | 20 | 55 | 0.9932 |
|  | 2001-2011 | 0.9472 | 30 | 65 | 0.9742 |
| Female | 1990-2001 | 0.9779 | 15 | 50 | 0.9832 |
|  | 2001-2011 | 0.9892 | 30 | 70 | 1.0038 |

Once inter-census coverage estimations were calculated they were linearly inter and extrapolated in order to have a yearly correction factor. This decision in based on the assumption that the rate of under-registration does not fluctuate widely but declines smoothly at a constant pace. One linear model for each sex gave us the yearly correction factor (CF). ${CF}_{males}= -1.562998+0.0013 year$ and ${CF}_{females}= -1.28424+0.0011 year$. In Graph 4, we displayed our life expectancy estimation using uncorrected and corrected death rates using the CF by sex.

Graph 4. Life expectancy estimations using corrected and uncorrected rates

**
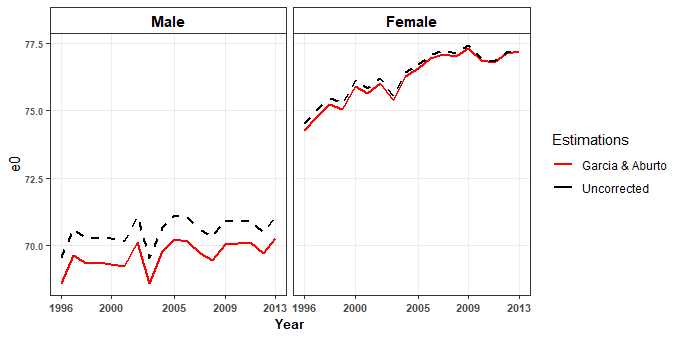
**

It is important to include in our correcting factors the continuous coverage improvements manifested during the period of our analysis. This is to avoid introducing artificial over-estimations corresponding to wider scope of the vital statistics system and not to demographic phenomena. Adjusting estimations by incorporating under-registration dynamics has become a mandatory task. We consider inter/extrapolating the inter-census under-registration ratios allowed us capturing theses change in the coverage and ensured accurate comparison through time. In this way, we avoid under-estimation at the beginning of the inter-census period and over-estimation of mortality due to adjusting through just one inter-census correction factor.

### **2.1.2 Infant mortality coverage**

Because there is not direct evaluation of the coverage of Infant Mortality Rate (IMR) estimations, we used indirect methods and compared the result obtained with the VMH’s death counts and different births counts estimations (WHO and VMH) including the one we considered the most appropriates for this analysis: ECLAC’s birth counts estimations. Additionally, we took information gathered on the last Census on the number of children ever born and children still alive by women’s age. This information is known as Summary Birth Histories (SBH). We used SBH to apply Brass’ model-based method to estimate IMR. Brass’ model-based method converts the proportions of children ever born and dead of women grouped by their age are into a standard life table function through adjusting fertility and mortality model-age patterns. The analysis was done considering an average exposure-time indirectly approached from mothers’ age.^(^[^26^](#_ENREF_26)^)^ Then, the probability of dying $q_{(0)}$depends on mother’s age ${(M}_{(x,5)}$ and the proportion of dead children ($D_{(x,5)}$)

$q_{(0)}=M_{(x,5)}*D_{(x,5)}$ (6)

We used Trussell’s variant of Brass method, which calculates multipliers to transform proportions of dead children into cohort–specific probabilities of dying through regression approach.^(^[^27^](#_ENREF_27)^)^ This method gives retrospective mortality information, so it is possible to estimate infant mortality levels for a period of about 15 years prior to the census. For all estimation, we considered west model of Coale-Demeny life tables. Results are displayed in Graph 5, a comparison with others international estimations is included in this graph.

Indirect estimations using Brass method were following the pattern found in IMR when considering VMH uncorrected data and ECLAC’s birth counts. Only last estimation for the year 2010 was lower than directly estimate IMR, this was because estimation on children survival for the group 15 to 19 years old women tends to be less accurate. Base on the results we kept IMR estimation with VMH’s death counts and ECLAC’s birth counts without any adjustment.

In comparison with others international IMR estimations, our IMRs capture clearly the critical period in Venezuela socio-political environment during the years 2002 to 2004, when two national strikes and a *coup d’état* took place. Even more, they evidenced the beginning of the slowing down on the improvements in infant mortality due to deterioration of health system, the shortage of medicines and the reduction of vaccination campaigns in many parts of the country.^(^[^28^](#_ENREF_28)^)^

**2.2 Quality of content**

We evaluate the proportion of missing or unknown information as well as the quality of age and sex of VMH’s death counts.

**2.2.1 Missing information**

The proportion of cases with unknown information in the variables sex, age and causes of death it is less than 0.35% for all the period considered. Yearly proportion of cases with unknown information are shown in Graph 6

*Graph 5. Venezuelan infant mortality rates by different method and data sources, 1996-2013*

Graph 6. Percentage of VHM’s death counts with missing information, 1996-2013.


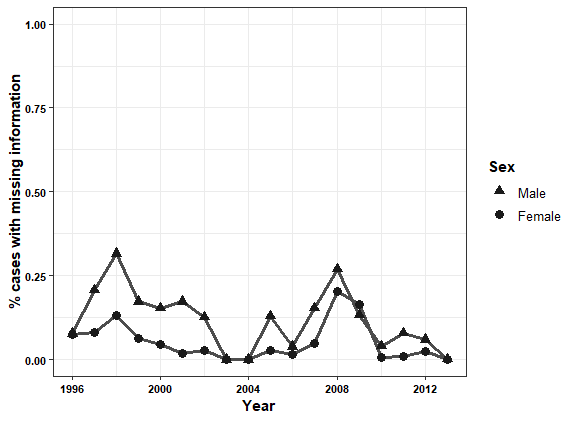


### **2.2.2 Age and sex declaration**

All demographic statistics are subject to errors, so evaluating the consistency and orderliness of the data by sex and age constitute a first step of the analysis. A literature review pointed out age heaping and age exaggeration as the main problems in mortality data in Latin America. We worked with aggregated data, which smoothed the effect of age heaping.

By its side, exaggerations tend to be concentrated in advanced ages.^(^[^29^](#_ENREF_29)^)^ To verify its existence, ratios of ten-year length cuts from population over 60 years were examined. Age ratios can provide a useful indication of possible undercounts or displacements between age groups. The higher are the ratios obtained the greater the chances of exaggerations in the declared age.^(^[^24^](#_ENREF_24)^)^ We fixed as limit the age ratios obtained from the Chilean death counts. Chilean population structure is identified as one of the most aged population structure in Latin America as well as one of the countries with better data quality. Ratios above Chilean would be indicative of age exaggeration in Mortality data. Results are displayed in Graph 7. Chilean ratios are represented in the blue line. Result showed 80-years-old-and-over male deaths during the 2000 and 2001 were the only cases in which the deaths ratios are over Chilean.

Graph 7. Age exagerattion ratios of death counts, 1996-2013.


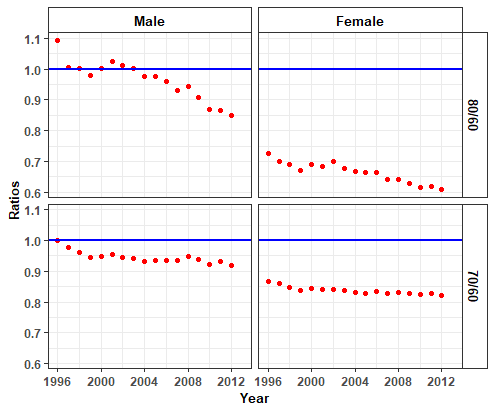


We also show sex ratios for all ages on VHM mortality data to evaluate the quality of sex declaration (Graph 8). Sex ratios in death counts turned out into the range of expected values, a bigger concentration of male death in young ages mostly due to high external causes of death. Lower levels of the ratios are concentrated just at the oldest ages.

Graph 8: VHM’s death counts sex ratios, 1996-2013.


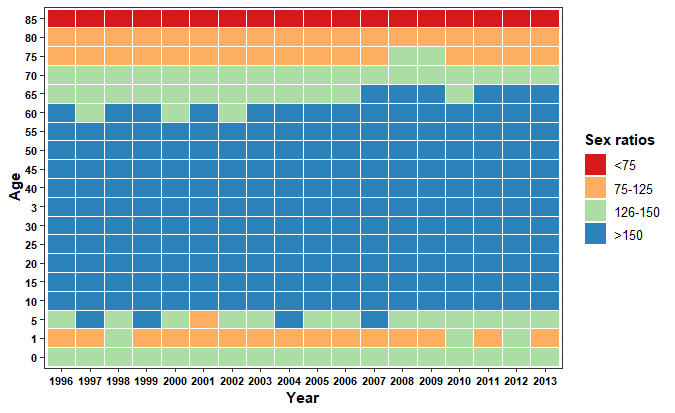


### **2.2.3. Ill-defined causes of deaths**

The percentage of cases classified as ill-defined causes was less than 2.5% at the end of the 90’s and continued declining until the year 2013 to less than 0.75% for both sexes. Yearly percentage of ill-defined causes of death in VHM data is shown in Graph 9.

Graph 9. Percentage of Ill-defined causes of death in VHM’s death counts, 1996-2013.


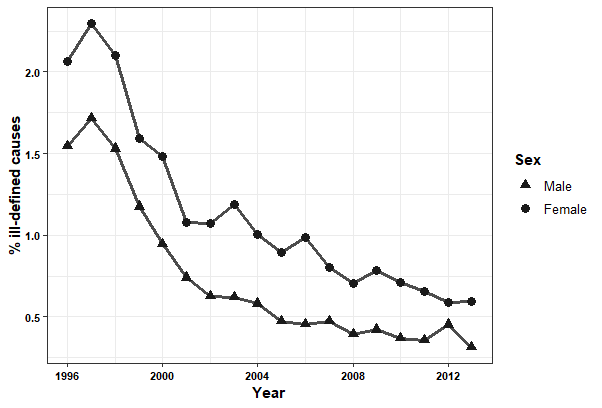


Cases reported as ill-defined causes of death and unknown age were incorporated into the data by using a proportional weights adjustment of the cases with completed information and defined causes of death. ^(^[^30^](#_ENREF_30)^)^

**3. Comparing with life expectancy estimations**

In this section, life expectancies using corrected specific mortality rates are compared with international estimations. Specifically: Global Burden of Diseases (GBD), United Nations Economic Commission for Latin American and the Caribbean (ECLAC), United Nations Population Division and Latin American Mortality Database (LAMBdA). All estimates were done using the same data sources but adjusted and calculated using different methods: ECLAC used long-term mortality rates for the period 1950 to 2015 and projected its pattern until 2050. This estimation is based on life tables implicit in the population projections. ECLAC has updated all their estimations in 2016 considering population reported during the last census in 2011. ^(^[^31^](#_ENREF_31)^)^

LAMBdA team produced inter-census adjusted life tables from vital statistics information. LAMBdA adjusted death rates for under-registration, adult age misstatement and ill-defined causes of death. Their adjustment procedures were chosen from a battery of 10-12 techniques that proved to be optimal in the sense of mean squared error reduction. ^(^[^32^](#_ENREF_32)^)^

Our estimates fall between ECLAC, LAMBDA and GBD’s estimates with overlapping confidence intervals, which makes us more confident about the robustness in our results. In general, all estimations follow the same trend for both sexes (Graph 10). Stagnation in male life expectancy is observed in all estimations as well as a continuous increase female life expectancy. Differences are seen in the level, being LAMBdA inter-census estimation the lowest and GBD annual estimations the highest. In 2006, LAMBdA life expectancy at birth is 67.6 years (male) and 74.9 years (female), while GBD estimation for the same year is 70.9 and 79.0 respectively. Our estimations are between GBD and LAMBDA and they are closer to those calculated by ECLAC updated in 2016.

Life expectancy shows a sudden decrease in 1999 when estimated by GBD, with a lower level than the others do. This is because for the year 1999, GBD included 30,292 deaths due to *exposure to forces of nature*. These deaths were attributed to a landslide occurred in the coast area of Vargas state. Indeed, initial unofficial reports given by local authorities rounded the death counts around 30,000 deaths during the days of the events. However, posterior specialized studies have showed that only 521 deaths could be officially attributable to the landslide, 333 in which the underlying cause of death was define as *exposure to forces of nature* in the mortality yearbook of that year. The others 188 deaths were registered on the mortality yearbook as ill-defined cause of death. Additionally to the registered deaths, only 331 people living on the area remained missing. ^(^[^33^](#_ENREF_33)^)^

Graph 10. Life expectancy at birth, comparison with different estimations


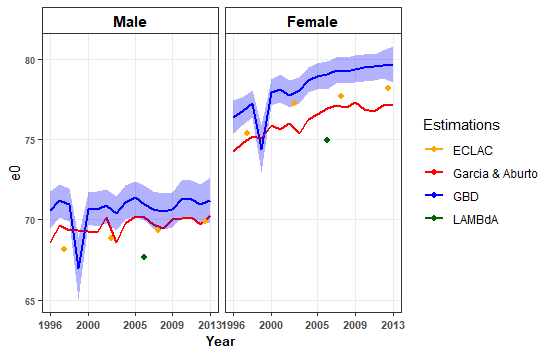


**4. Causes of death classification**

Table 3. Causes-of-death groups and ICD codes

| **Causes of death group** | **International Classification of Diseases**  **10th revision coding** |
| --- | --- |
| Circulatory diseases (cardiovascular, stroke) | I05-I09, I11, I13, I21-I51, I60-I69 |
| Neoplasms | C00-C97 |
| Diabetes | E10-E14 |
| Homicides and other violent causes with undetermined intention | X85-Y09, Y10-Y34, Y35-Y36 |
| Other external causes (including traffic accidents, injuries and suicide) | V01-V89, V90-X59, X60-X84 |
| Respiratory Diseases | J00-J98 |
| Infectious Diseases | A00-B99 |
| Digestive Diseases | K00-K92 |
| Conditions originated in the perinatal period | P00-P96 |

**5. Trends in violent causes of death**

Trends on violent deaths by sex are show in Graph 11. Additionally, age-specific violent rates by sex estimations 1996 to 2013 are compared with Global Burden of Diseases (GBD) estimations (Graph 12 for male and Graph 13 for females). Our corrected estimations show slightly higher rates than GBD estimations for male at all ages and females between 15 and 24 years. It is likely that our estimates are higher and more accurate because we applied a coverage correction; this decision is due to several sources have documented the undercounting of deaths in Venezuela, including the World Health Organization. In addition, we defined violent deaths as those in which the death certificate stablished ‘Homicide’ as primary cause of death *(Assault*: X85-Y09) but also *Event of undetermined intent* (Y10-Y34) and *Legal intervention and operations of war* (Y35-Y36). This is important because some homicides are usually not counted if the intent is not legally determined. The legal determination depends highly on the police’s investigation and not to the actual occurrence.

**6. Brief description of the lifespan variation indicator**

In lifetable notation, it is:

$$\sigma=\sqrt{\int_{a}^{\omega} {(x-e_{a})}^{2}f\left( x \right)\text{d}x}. (7)$$

Where $f\left( x \right),e_{0}$ and $\omega$ denote the age at death density function, life expectancy at age $a$, and the open-aged interval (85+ in our case), respectively.

Graph 11. Age-specific homicide rates by sex in Venezuela


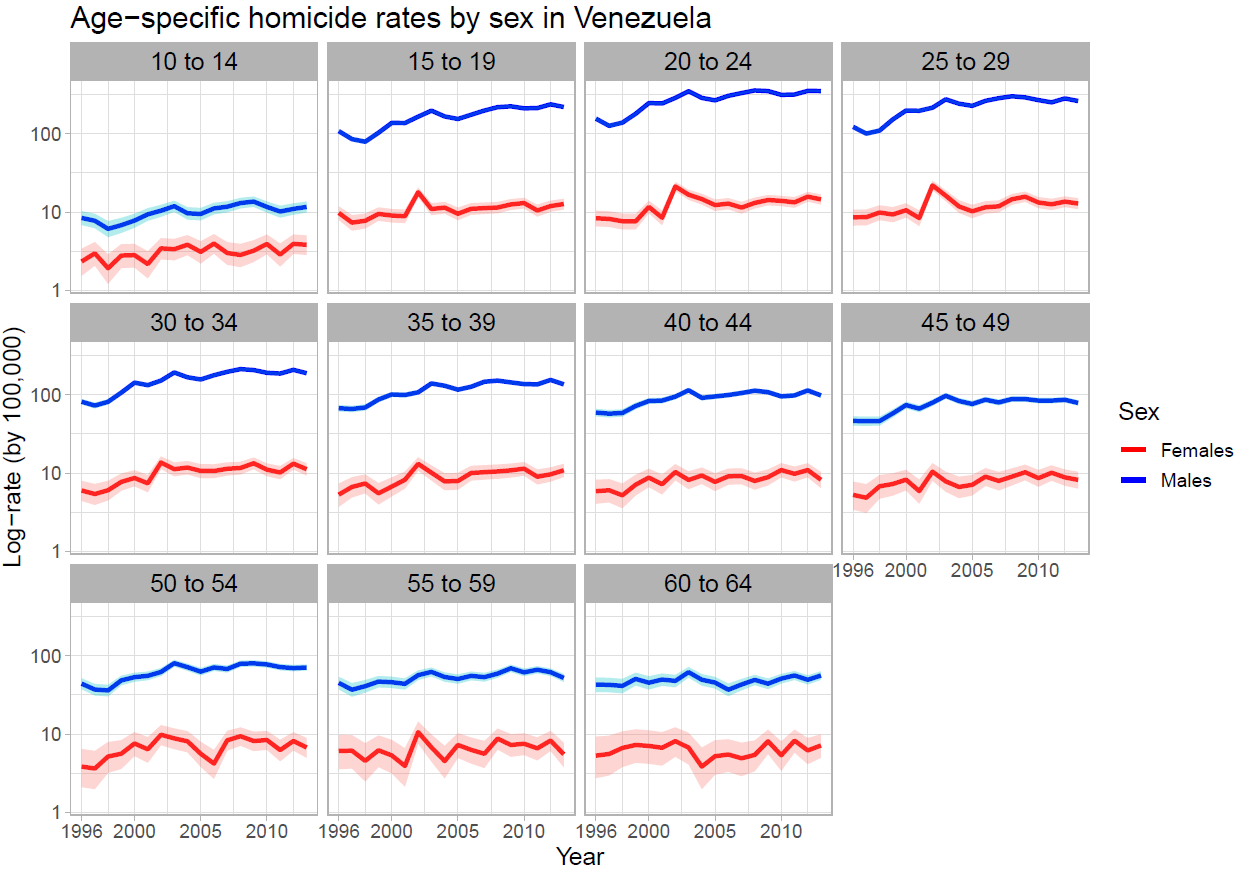


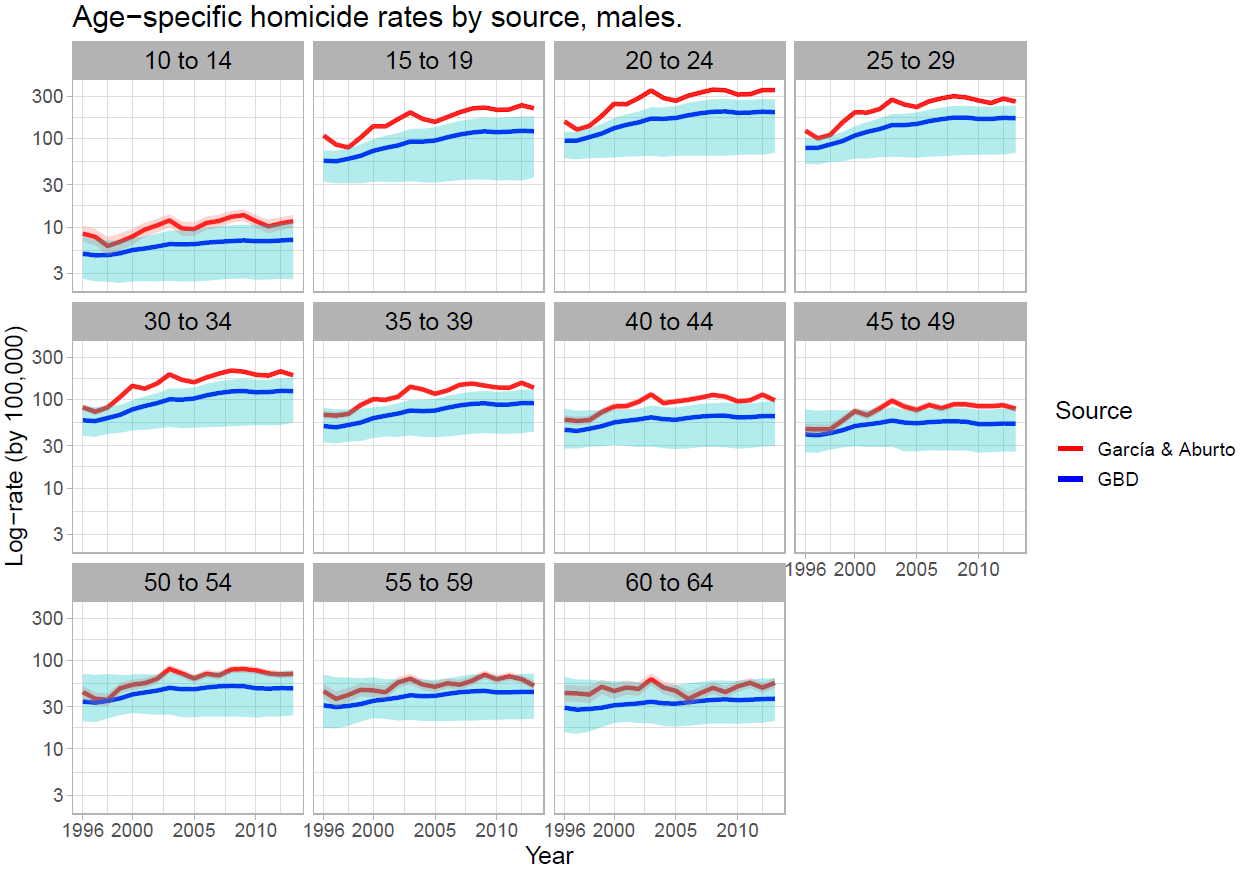


Graph 12. Age-specific violent male deaths by source, 1996-2013

Graph 13. Age-specific violent female deaths by source, 1996-2013.


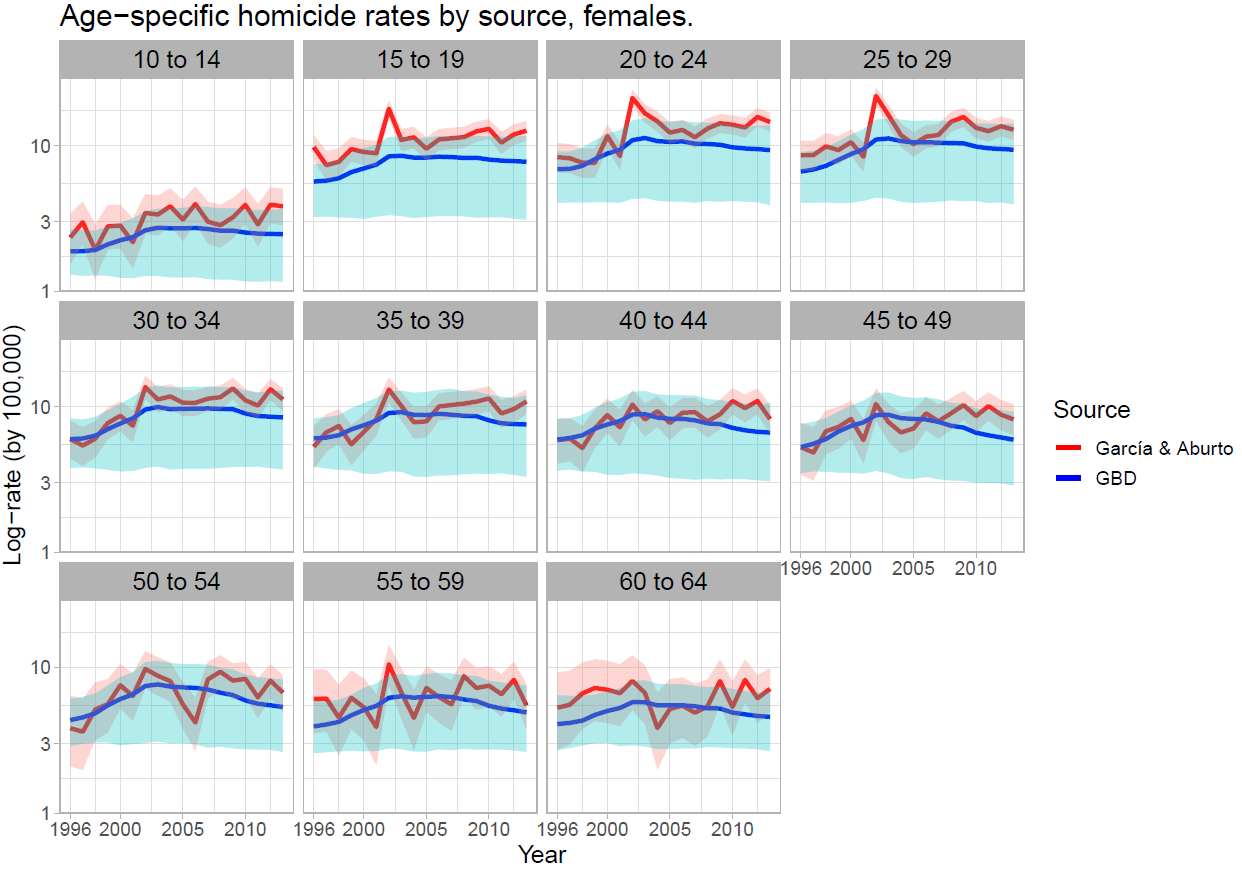


**7. Description of the decomposition method**

The decomposition method used in this paper is based on the line integral model (Horiuchi et al 2008). Suppose $f$ (e.g. $e^{\dagger}$ or life expectancy) is a differentiable function of $n$ covariates (e.g. each age-cause specific mortality rate) denoted by the vector $\mathbf{A}=[x_{1},x_{2}, \ldots,x_{n}]^{T}$. Assume that $f$ and $\mathbf{A}$ depend on the underlying dimension$t$, which is time in this case, and that we have observations available in two time points $t_{1}$ and $t_{2}$. Assuming that $\mathbf{A}$ is a differentiable function of $t$ between $t_{1}$ and $t_{2}$, the difference in $f$ between $t_{1}$ and $t_{2}$ can be expressed as follows:

$$f_{2}-f_{1}= \sum_{i=1}^{n} \int_{x_{i}\left( t_{1} \right)}^{x_{i}\left( t_{2} \right)} \frac{\partial f}{\partial x_{i}}dx_{i}=\sum_{i=1}^{n} c_{i}, (8)$$

where $c_{i}$ is the total change in $f$ (e.g. $e^{\dagger}$ or life expectancy) produced by changes in the $i$-th covariate, $x_{i}$. The $c_{i}$'s in equation (2) were computed with numerical integration following the algorithm suggested by Horiuchi et al (2008). This method has the advantage of assuming that covariates change gradually along the time dimension.

**8. Wealth and inequality in Venezuela**

Continuous economic growth combines with reducing income inequality managed to lift an important proportion of Venezuelans out of poverty, to the extent that Venezuela became the least unequal Latin American country from 2006 to 2011 in terms of Gini coefficient. ^(^[^31^](#_ENREF_31)^)^ Increases of the annual gross domestic product (GDP) per capita and reducing Gini coefficient for the period are shown in Graph 14. As consequence, reduction of the proportion of population living in extreme poverty were reported as well as the perception of not to earn enough income (Graph 15).

Graph 14. Annual Gross Domestic Product (GDP) per capita and Gini coefficient, 1996-2013


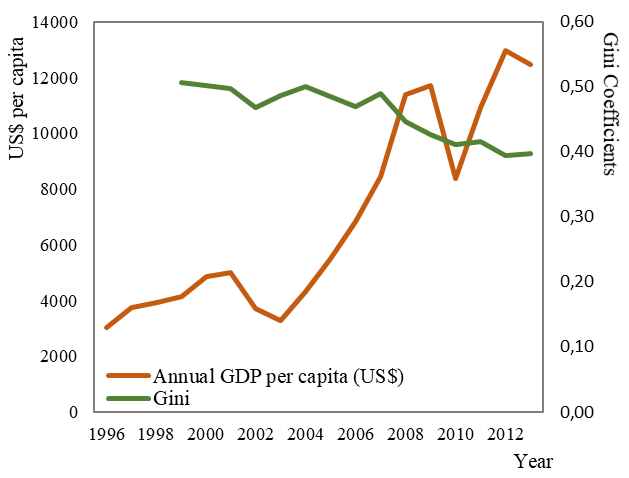


Source: Economic Commission for Latin America and the Caribbean- Statistics and Economic Projections

Division, Social Statistics Unit. Based on special tabulations of the Latinobarometro Corporation Survey

Graph 15. Percentage of population living in extreme poverty and population declaring not to have enough incomes,1996-2013


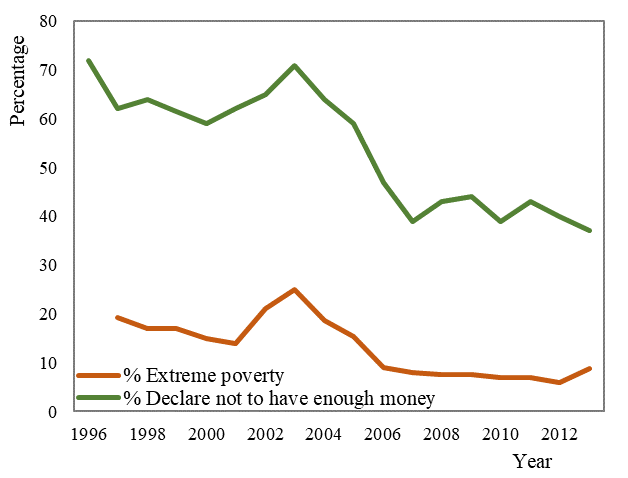


Source: Economic Commission for Latin America and the Caribbean- Statistics and Economic Projections

Division, Social Statistics Unit. Based on special tabulations of the Latinobarometro Corporation Survey

**9. Crime in Venezuela**

Crime has risen sharply in the period. Reported kidnapping cases is one example of how crime has augmented at the same time than incomes (Graph 16). Victimization rate -also displayed in Graph 16- measured as the number of persons reporting to have being victims or having a family member who has been victim of a crime during the last year, showed increase trends.

Graph 16. Victimization rate and number of reported kidnapping cases


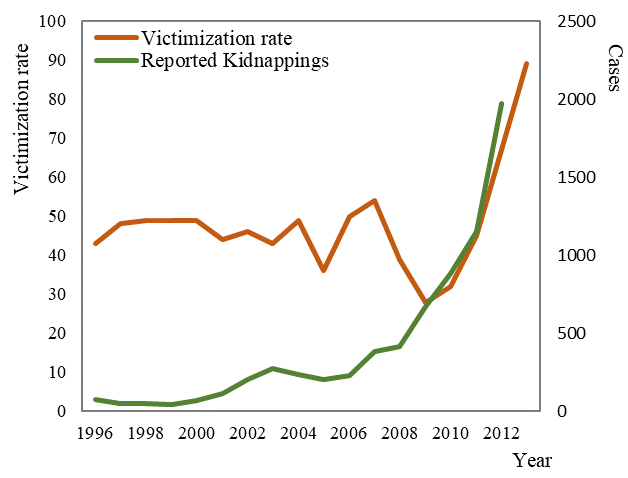


Source: Statistics and Economic Projections Division, Social Statistics Unit. Based on special tabulations of the Latinobarometro Corporation Survey and reported kidnappings cases to the Venezuelan Bureau of Scientific and Criminal Investigations (VBSCI)

# **Reference**

1. VMH. Mortality Yearbooks 1990-2013. In: Health VMo, editor. Caracas: Epidemiology Division; 2014.

2. VNIS. Registered death counts 1990-2012. In: Statistics NIo, editor. Caracas: Vital Statistics Department; 2016.

3. PAHO. Basic indicators. Washigton: Pan-American Health Organization, 2014.

4. Bay G, Orellana H. La calidad de las estadísticas vitales en la América Latina. Expert workshop on the use of vital statistics: scope and limitations; December 13th and 14th; Santiago Economic commission for Latin American and the Caribbean; 2007.

5. Bay G. América Latina: diferencia relativa de las defunciones totales (por cien) implícitas en las estimaciones y proyecciones de población y las defunciones registradas y disponibles según quinquenio, 1950-2005. Santiago de Chile: Economic Commission for Latin American Countries and The Caribbean, 2015.

6. The Lancet Editorial. The collapse of the Venezuelan health system. The Lancet. 2018;391:1331.

7. Fraser B. Data reveal state of Venezuelan health system. The Lancet. 2017;389(10084):2095.

8. Briceño-León R. Violence in Venezuela: oil rent and political crisis. Ciência & Saúde Coletiva. 2006;11:315-25.

9. UNODC. Global study on homicide 2013. Vienna: United Nations Office on Drugs and Crime, 2014 Contract No.: 4.

10. VNIS. Long-term population estimations. In: Statistics VNIo, editor. Caracas: Demographic Division; 2013.

11. Tacla Chamy O. La omission censal en America Latina, 1950-2000. Santiago de Chile: United Nations Economic Commission for Latin America and the Caribbean, 2006.

12. UN. Trends in International Migrant Stock: Migrants by Destination and Origin (United Nations database, POP/DB/MIG/Stock/Rev.2015). In: United Nations DoEaSA, editor. 2015.

13. ECLAC. REtrieval of DATa for small Areas by Microcomputer REDATAM (Revision 06). Santiago de Chile: United Nations Economic Commission ofr Latin America and the Caribbean; 2011.

14. ECLAC. Investigacion de la Inmigracion Internacional en America Latina (IMILA). In: Caribbean UNECfLAat, editor. Santiago de Chile: Population Division; 2000.

15. ECLAC. the Internal Migration in Latin America and the Caribbean database (MIALC) In: Caribbean UNECfLAat, editor. Santiago de Chile: Population Division; 2008.

16. ECLAC. Indigenous and Afro-descendant Population in Latin America and the Caribean database (PIAALC) In: Caribbean UNECfLAat, editor. Santiago de Chile: Population Division; 2016.

17. ECLAC. Urbanization and Spatial Population Distribution in Latin America and the Caribbean database (DEPUALC) In: Caribbean UNECfLAat, editor. Santiago de Chile: Population Division; 2016.

18. ECLAC. Principales cambios en las boletas de los censos latinoamericanos de las décadas de 1990, 2000 y 2010. Santiago de Chile: Economic Comission for Latin American and Caribbean, 2012.

19. ECLAC. Population Projection. Santiago de Chile: United Nations Economic Commission for Latin Amrican and the Caribbean, 2011.

20. VNIS. Household and population census 2011. REDATAM micro-data [Online]. In: Statistics VNIo, editor. 2011.

21. VMH. Maternal and Infant Mortality, Bolivarian Republic of Venezuela, 1990-2009. In: Health VMo, editor. Caracas: Office of Health Situation Analysis; 2012.

22. VNIS. Vital Statistics Query System. . Birth counts. [Online] 2000-2012. In: Statistics NIo, editor. Caracas: Vital Statistics Division; 2014.

23. ECLAC. Bolivarian Republic of Venezuela: Long-term population estimations and projections, 2016's revision. In: Caribbean UNECfLAat, editor. Santiago de Chile: CELADE; 2017.

24. Agostinho C. Estudo sobre a mortalidade adulta, para Brasil entre 1980 e 2000 e Unidades da Federação em 2000: uma aplicação dos métodos de distribuição de mortes. Belo Horizonte: Universidade Federa Minas Gerais; 2009.

25. Palloni A, Pinto-Aguirre G. Adult Mortality in Latin America and the Caribbean. In: Rogers RG, Crimmins EM, editors. International Handbook of Adult Mortality. Dordrecht: Springer Netherlands; 2011. p. 101-32.

26. UN. Manual x técnicas indirectas de estimación demográfica. New York: United Nations Population Division 1986.

27. Dorrington R. The Synthetic Extinct Generations method. In: Moultrie T, Dorrington R, Hill A, Hill K, Timæus I, Zaba B, editors. Tools for Demographic Estimation. Paris: International Union for the Scientific Study of Population; 2013.

28. VEAPHR. The right to health in Venezuela. Caracas: Venezuelan Education-Action Program on Human Right, 2018.

29. Coale AJ, Kisker EE. Mortality Crossovers: Reality or Bad Data? Population Studies. 1986;40(3):389-401.

30. Shryock E, Siegel J. The Methods and Materials of Demography. ediction Fp, editor. Washigton DC: US Bureau of the census; 1980 June 1980.

31. ECLAC. CEPALSTAT Database: Technical notes. In: Caribbean UNECfLAat, editor. Santiago de Chile2016.

32. Palloni A, Pinto G, Beltrán-Sánchez H. Latin American Mortality Database (LAMBdA). In: Wisconsin Uo, editor. Madison, Wisconsin2014.

33. Altez R. Muertes bajo sospecha: Investigación sobre el número de fallecidos en el desastre del estado Vargas, Venezuela, en 1999. Cuadernos de Medicina Forense. 2007:255-68.
